# The Computational Diet: A Review of Computational Methods Across Diet, Microbiome, and Health

**DOI:** 10.3389/fmicb.2020.00393

**Published:** 2020-04-03

**Authors:** Ameen Eetemadi, Navneet Rai, Beatriz Merchel Piovesan Pereira, Minseung Kim, Harold Schmitz, Ilias Tagkopoulos

**Affiliations:** ^1^Department of Computer Science, University of California, Davis, Davis, CA, United States; ^2^Genome Center, University of California, Davis, Davis, CA, United States; ^3^Department of Microbiology, University of California, Davis, Davis, CA, United States; ^4^Process Integration and Predictive Analytics (PIPA LLC), Davis, CA, United States; ^5^Graduate School of Management, University of California, Davis, Davis, CA, United States

**Keywords:** microbiota, gut microbiome, machine learning, artificial intelligence, data analytics, nutrition

## Abstract

Food and human health are inextricably linked. As such, revolutionary impacts on health have been derived from advances in the production and distribution of food relating to food safety and fortification with micronutrients. During the past two decades, it has become apparent that the human microbiome has the potential to modulate health, including in ways that may be related to diet and the composition of specific foods. Despite the excitement and potential surrounding this area, the complexity of the gut microbiome, the chemical composition of food, and their interplay *in situ* remains a daunting task to fully understand. However, recent advances in high-throughput sequencing, metabolomics profiling, compositional analysis of food, and the emergence of electronic health records provide new sources of data that can contribute to addressing this challenge. Computational science will play an essential role in this effort as it will provide the foundation to integrate these data layers and derive insights capable of revealing and understanding the complex interactions between diet, gut microbiome, and health. Here, we review the current knowledge on diet-health-gut microbiota, relevant data sources, bioinformatics tools, machine learning capabilities, as well as the intellectual property and legislative regulatory landscape. We provide guidance on employing machine learning and data analytics, identify gaps in current methods, and describe new scenarios to be unlocked in the next few years in the context of current knowledge.

## Introduction

**FIGURE 1:**
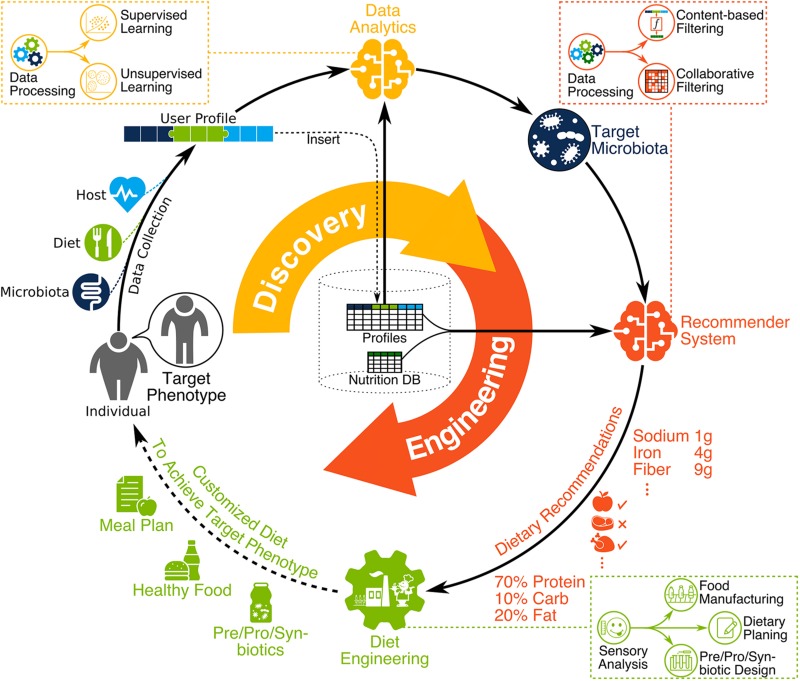
The vision for the next nutrition revolution involves microbiome-aware dietary planning and manufacturing. First, DGMH data is collected, homogenized, and stored, with any new user data integrated as part of a cohesive compendium. Then, DGMH data are analyzed (*data analytics*) to identify the functional characteristics and target microbiota, personalized to the individual and the desired phenotype. This includes data processing followed by supervised and unsupervised learning using a user profile compendium. Bioinformatic tools are used during data processing to extract meaningful information from raw high-throughput data such as metagenomic sequence reads. Then, the *recommendation system* provides dietary recommendations to help achieve target microbiota. This includes the integration of user profiles in a compendium along with nutrition DB proceeded by data processing then content-based and collaborative filtering. Finally, *diet engineering* is performed to create dietary products for the user. This includes the design of prebiotics, probiotics, synbiotics, manufactured food, and detailed dietary planning. In practice, taste and flavor of dietary products is very important to help users commit to any given diet, therefore sensory analysis should inform all dietary engineering efforts.

During the past two decades, the human microbiome has emerged as a biological system with the potential to significantly influence health and disease (Shreiner et al., 2015). Despite our limited understanding regarding its intricate relationship with the host and its environment (Foster et al., 2017), recent discoveries related to the human microbiome have opened new horizons in food science (Barratt et al., 2017), precision medicine (Wishart, 2016), and biotechnology (Taroncher-Oldenburg et al., 2018) among other fields. In parallel, advances in genomics and bioinformatics have provided inexpensive tools to acquire biological and clinical data, as well as the tools to translate the data into knowledge (Shoaie et al., 2015; Zeevi et al., 2015; Thaiss et al., 2016a; Korem et al., 2017; Baldini et al., 2018; Bauer and Thiele, 2018; Gilbert et al., 2018; Greenhalgh et al., 2018; Knight et al., 2018). Given these advances, the integration of diet, gut microbiome, and human health (DGMH) data has the potential to drive a paradigm shift in the way wellness states are measured, diseases are treated, products are designed, and health interventions are administered. To realize this potential, advances in knowledge are required in order to optimize the composition and metabolic dynamics of microbial communities in relation to desired health and performance outcomes—from dietary interventions and bioengineered products to lifestyle changes and the living environment (Figure 1).

In this article, we summarize the research that has been done related to DGMH, with a focus on DGMH data and computational methods. We begin with a brief overview of key areas of current knowledge regarding the interaction between diet, health, and the gut microbiome. We then proceed to review the available data sources and the computational methods currently used, investigate the role that machine learning and artificial intelligence (AI) can play in this area, and summarize the intellectual property (IP) and legislative regulatory landscape. We conclude with recommendations to accelerate research and development efforts through better integration of research resources and tools, especially in the context of computational science and data analytics. A glossary of terms is provided in Table 6.

In general, the most recent articles reviewing the computational tools for microbiome data focusing on metagenomic data processing methods provide limited guidance on employing machine learning and data analytics and do not furnish recommendations in the context of DGMH data. The purpose of this manuscript is to help fill this gap by considering relevant literature, describing key challenges and potential solutions, and proposing a framework to improve the potential for research initiatives to accelerate progress in this exciting and potentially revolutionary field.

### Current Knowledge: Gut Microbiota and Human Health

Emerging evidence suggests that the intestinal microbiota plays a significant role in modulating human health and behavior [see comprehensive reviews (Sherwin et al., 2018; Pereira et al., 2019; Zmora et al., 2019)]. Several studies have demonstrated that the human intestinal microbiota is seeded before birth (Stinson et al., 2019), and the mode of delivery influences the composition of the gut microbiota (Ferretti et al., 2018; Shao et al., 2019). The gut of a vaginally born newborn is enriched primarily with the vaginal microbiota from the mother, while a cesarean procedure results in the newborn’s gut microbiota being dominated by the microbiota of the mother’s skin as well as points of contact at the hospital (Dominguez-Bello et al., 2010). Microbial diversity is very dynamic during infancy and increases and converges to an adult-type microbiota by 3–5 years of age (Rodríguez et al., 2015). Evidence is also building to suggest that diet plays a key role in shaping the composition of microbial communities in the infant’s gut. For example, species of beneficial bacteria such as *Lactobacillus* and *Bifidobacterium* have been found to be dominant in breastfed infants while species of harmful bacteria such as *Clostridium*, *Granulicatella*, *Citrobacter*, *Enterobacter*, and *Bilophila* have been found to be dominant in formula-fed infants (Bäckhed et al., 2015). In addition, breastfed babies have higher gut microbial diversity compared to formula-fed babies, and several studies indicate that the diversity of bacteria is directly connected to health (Wang et al., 2008; Bäckhed et al., 2015). An unbalanced composition of the infant’s gut microbiota has been linked to several childhood diseases, including atopic dermatitis (AD) (Abrahamsson et al., 2012; Zheng et al., 2016) obesity (Yuan et al., 2016), and asthma (Thavagnanam et al., 2008).

**FIGURE 2:**
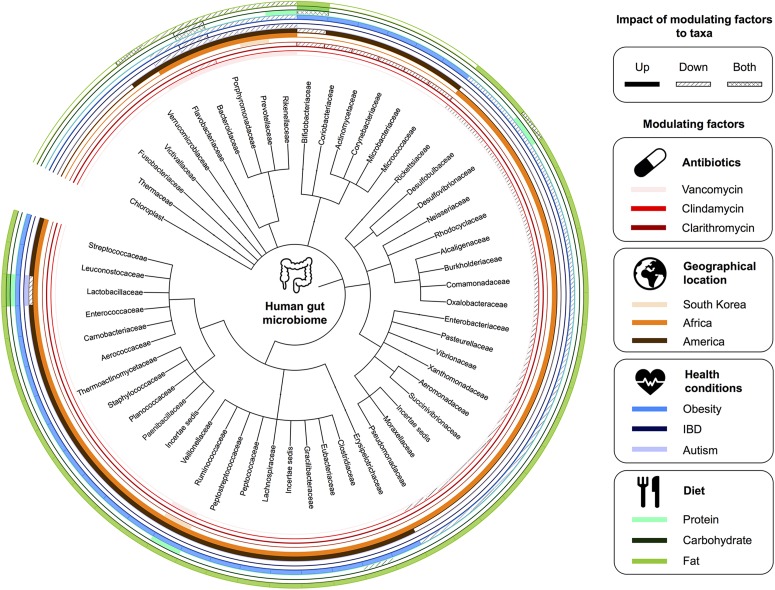
Factors affecting the gut microbiota. A summary of human gut microbiome taxonomy at the family level and the corresponding modulating factors.

The composition of the gut microbiota of an adult human is relatively stable (Shreiner et al., 2015), but several factors can influence it, including antibiotic treatment, long-term change in diet, microbial infections, and lifestyle (Willing et al., 2011; Conlon and Bird, 2015; Mathew et al., 2019; Zmora et al., 2019). Several health conditions are linked to changes in a stable and established gut microbiota such as Crohn’s disease (Manichanh et al., 2006), psoriatic arthritis (Scher et al., 2015), type 1 diabetes (de Goffau et al., 2013), atopic eczema (Wang et al., 2008), celiac disease (Schippa et al., 2010), obesity (Castaner et al., 2018), type 2 diabetes (Qin et al., 2012), and arterial stiffness (Menni et al., 2018). However, further research is required to establish direct links between these conditions and the composition of microbial communities in the gut. Interventions, such as oral administration of probiotics/prebiotics and fecal transplants, have shown efficacy on reducing the severity of some conditions, such as diarrhea, acute upper respiratory tract infections, eczema, Crohn’s disease, and ulcerative colitis (Anderson et al., 2012; Mansfield et al., 2014; Hao et al., 2015; Saez-Lara et al., 2015; Goldenberg et al., 2017; Delzenne et al., 2019). See Figure 2 for illustration of factors affecting the gut microbiota.

### Data

**FIGURE 3:**
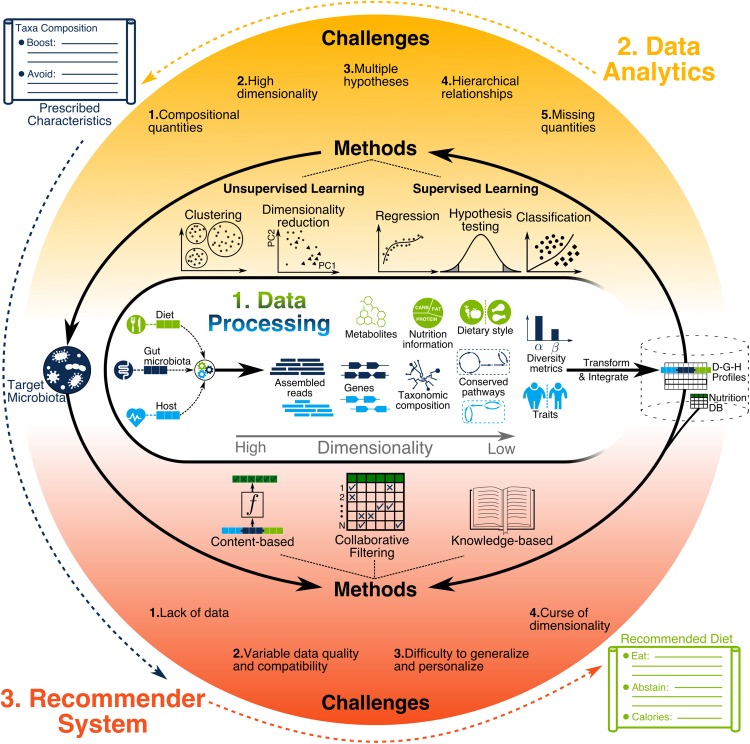
Illustration of data processing, data analytics, and recommendation systems. Data processing generates diverse types of information with different levels of resolution and dimensionality. Such information needs to be transformed and integrated across all users for building a compendium. Next, data analytics methods are used to discover the characteristics of target microbiota prescribed for individuals to achieve their health objectives. Finally, recommendation system methods use the compendium to find the dietary recommendations for helping individuals achieve the target microbiota.

The increase in size and heterogeneity of information gathered by microbiome studies present great opportunities and serious data analysis challenges (Wooley et al., 2010), with many tools developed to address them (Breitwieser et al., 2017; Quince et al., 2017). These bioinformatics tools quantify low dimensional biological variables, such as the relative abundance of microbial species and metabolites, by using high dimensional data such as DNA sequence reads and mass spectrometry (MS) signatures as illustrated in Figure 3. Depending on data quality, sample size, and research hypothesis, different information dimensionalities are used, such as gene-level (Vatanen et al., 2018) or functional gene ontology terms (Brown et al., 2011).

#### Gut Microbiota Data

Functional characteristics of microbial communities can be revealed using high-throughput metametabolomics (Walker et al., 2014) and metaproteomics (Verberkmoes et al., 2009; Zhang et al., 2018) using MS technologies. Metagenomic and metatranscriptomic content of gut microbiota (which give rise to the functional characteristics) can be quantified using DNA sequencing. The most widely used approach for gut microbiota profiling is *marker gene sequencing*, which relies on sequencing counts of the hypervariable 16S genes to calculate Operational Taxonomic Units (OTUs) (Amann et al., 1995). Searching OTUs against reference databases such as Greengenes (McDonald et al., 2012) and SILVA (Quast et al., 2012) allows inferring relative taxa abundances in a microbiome sample (Langille et al., 2013). *Whole-genome or shotgun metagenomics* (Quince et al., 2017) is a recent technique that not only reveals the microbial community structure, but it can also quantify relative abundances of genes, taxa, conserved functional groups, or over-represented pathways. Within-sample (alpha) and cross-sample (beta) diversity of microbiome can be calculated with respect to genetic, taxonomic, functional, or metabolic pathway profiles of samples (Turnbaugh et al., 2009; Martiny et al., 2011; Huttenhower et al., 2012; Lozupone et al., 2012; Heintz-Buschart and Wilmes, 2017; Ranjan et al., 2018). The Shannon index, Chao1, and Abundance-based Coverage Estimator (ACE) are used to measure alpha diversity while UniFrac, weighted UniFrac, and Bray–Curtis calculate beta diversity. In longitudinal studies, the same measures of diversity, or more sophisticated eigenvalue-based analyses, can quantify the microbiota stability across timepoints (Lozupone et al., 2012; Relman, 2012; Coyte et al., 2015; Mehta et al., 2018). Jackknifing and bootstrapping are used to estimate the bias in diversity estimates, particularly when estimating the number of species (i.e., species richness) in samples (Smith and van Belle, 1984). Some of the most significant publicly available microbiome datasets are listed in Table 1.

**TABLE 1:** Publicly available data from gut microbiota studies.

Project, database, or repository name	Number of cases	Sample types	Disease related (Y/N/B)	Data availability (Y/N/Conditional)	Website
Human Microbiome Project (HMP1)	300	Nasal passages, oral cavity, skin, gastrointestinal tract, and urogenital tract	N	Y	NIH Human Microbiome Project - Home, 2019
Integrative Human Microbiome Project (iHMP): pregnancy and preterm birth (MOMS-PI)	∼2,000	Mouth, skin, vagina, and rectum	Y	Y	NIH Human Microbiome Project - Home, 2019
Integrative Human Microbiome Project (iHMP): onset of IBD (IBDMDB)	∼90	Stool and blood	Y	Y	NIH Human Microbiome Project - Home, 2019
Integrative Human Microbiome Project (iHMP): onset of type 2 diabetes (T2D)	∼100	Fecal, nasal, blood, serum, and urine	Y	Y	NIH Human Microbiome Project - Home, 2019
American Gut Project (AGP)	>3,000	Stool and swabs from skin/mouth	B	Y	American Gut, 2019
Personal Genome Project microbiota component (PGP)	>5,000	Skin/oral/fecal	−	Y	Data – The Harvard Personal Genome Project (PGP), 2019
TwinsUK	>11,000	Multiple	−	C	TwinsUK, 2019
Global Gut Project (GG)	531	Fecal	N	Y	Yatsunenko et al., 2012
Project CARDIOBIOME	>4,000	−	−	N	
Pediatric Metabolism and Microbiome Repository (PMMR)	∼350	Human microbial cell lines, stool, and/or DNA and RNA	Y	N	https://clinicaltrials.govClinicalTrials.gov, 2019
Lung HIV Microbiome Project (LHMP)	162	Lung, nasal, and/or oropharyngeal cavities	Y	Y	BioLINCC, 2019
The Study of the Impact of Long-Term Space Travel on the Astronauts’ Microbiome (Microbiome)	9	Saliva and gastrointestinal	N	N	NASA, 2019
Michigan Microbiome Project (MMP)	−	−	−	N	The Michigan Microbiome Project, 2019
uBiome	−	Gut, mouth, nose, genitals, and skin	B	C	
Human Oral Microbiome Database (eHOMD)	−	Upper digestive and upper respiratory tracts, oral cavity, pharynx, nasal passages, sinuses, and esophagus	−	Y	HOMD : Human Oral Microbiome Database, 2019
Human Pan-Microbe Communities (HPMC)	>1,800	Gastrointestinal	B	Y	HPMCD: Human Pan Microbial Communities Database, 2019
Curated Metagenomic Data	>5,000	Multiple	B	Y	curatedMetagenomicData, 2019
European Nucleotide Archive	−	−	−	Y	European Nucleotide Archive EMBL-EBI, 2019
EBI-metagenomics portal samples	>20,000	Multiple	B	Y	EMBL-EBI Mg, 2019
MG-RAST	>10,000	Multiple	B	Y	MG-RAST, 2019

#### Diet Data

Various types of dietary information are collected in gut microbiome studies. This includes fine-grain information such as mass spectrometry (MS) signatures and metagenomic reads (Quinn et al., 2016) or coarse grain information such as dietary style [e.g., Western vs. Mediterranean diet (De Filippis et al., 2016)] from study participants. Diet data collection is often questionnaire-based, either through self-reporting or by a trained interviewer. For self-reporting, a food frequency questionnaire (FFQ) and 24-h dietary recall (24HR) can be used where participants report their dietary intake either every 24 h or over a longer period through a checklist of food items (Shim et al., 2014). A dietary record (DR) can also be used where data collection is done when food is consumed (e.g., using smartphones),which minimalizes reliance on participant’s memory. After data collection, the intake amount of macronutrients (fat, carbohydrates, and protein), micronutrients (vitamins and minerals), and food metabolites can be estimated by querying the food items against food composition databases such as the USDA food composition database (US Department of Agriculture and Agricultural Research Service, 2010) and the Canadian nutrient file (Canada, 2010). Note that microbiota of dietary intake can be characterized using metagenomic sequencing as reviewed previously, if not already defined [e.g., probiotics with predefined strains (Sánchez et al., 2017)]. Some studies perform metabolic characterization of dietary intake directly (Quinn et al., 2016), while others rely on pre-characterized metabolic profiles (Zhao et al., 2018). A significant limitation of any analysis is that food composition databases characterize only 0.5% of the known nutritional compounds (Barabási et al., 2019).

#### Host Data

Profiled host information types can be very high dimensional [e.g., high-throughput genome sequences (Hall et al., 2017)] or low dimensional [e.g., obese vs. non-obese (Thaiss et al., 2014; Cox and Blaser, 2015)]. Host genotype data can come from whole-exome sequencing (WES) (Gopalakrishnan et al., 2018) or a genome-wide association study (GWAS) (Bonder et al., 2016; Turpin et al., 2016). It can also be extended by predicting the whole-genome sequence for each individual through genotype imputation software (Howie et al., 2009), as done in several studies (Bonder et al., 2016; Goodrich et al., 2016; Rothschild et al., 2018). Host transcriptomic profiles can be assessed directly using microarrays (Schwartz et al., 2012; de Steenhuijsen Piters et al., 2016) and RNA-Seq (Thaiss et al., 2016b; Pan et al., 2018) or imputed using tools such as PrediXcan (Gamazon et al., 2015) with GWAS data. The genetic and transcriptomic profiles can be summarized into informative lower-dimensional features through gene ontology categories and metabolic pathways using databases such as MetaCyc (Caspi et al., 2017), KEGG (Kanehisa et al., 2011), Reactome (Fabregat et al., 2017), or GO (Antonazzo et al., 2017). Today, limited microbiome studies perform such analysis (Blekhman et al., 2015; Davenport et al., 2015; Dobson et al., 2015). Other important information such as age, gender, ethnicity, body weight, blood pressure, dietary restrictions, and diseases of a host organism can be extracted from medical records, surveys, and interviews.

## Computational Analysis

There have been various reviews concerning microbiome data processing and analysis (Tyler et al., 2014; Tsilimigras and Fodor, 2016; Breitwieser et al., 2017; Quince et al., 2017; Knight et al., 2018). Here we focus on data analytics, machine learning, and AI-based recommendation system methods that enable microbiome-aware systems involving diet and wellness. We provide readers insight into important methods, challenges that arise, suggested solutions as well as blueprints of example scenarios to be used in their research. See Qu et al. (2019), Topçuoglu et al. (2019), and Zhou and Gallins (2019) for further explanation and examples of the machine learning methods discussed here.

### Microbiome Data Processing Tools

There are a substantial number of publicly available microbiome data processing methods and pipelines that can generate the various types of data discussed. Table 2 provides a representative summary of such methods and pipelines. For 16S data, QIIME (Caporaso et al., 2010) and MOTHUR (Schloss et al., 2009) provide a wider range of options for the user compared to UPARSE (Edgar, 2013), but all are popular pipelines. QIIME 2 (Bolyen et al., 2019) is now emerging as a powerful replacement to its predecessors, partly due to its extensibility and support. For whole metagenomic sequencing, methods such as Kraken (Wood and Salzberg, 2014), MEGAN (Huson et al., 2016), MetaPhlAn2 (Truong et al., 2015), and HUMAnN (Abubucker et al., 2012) are used.

**TABLE 2:** A summary of highlighted methods and pipelines for microbiome data processing.

Steps	Sub-step descriptions	Highlighted methods and their availability in popular pipelines (QIIME, MOTHUR, and UPARSE)
(1) Quality control	Chimera removal and noise mitigation	Trimmomatic^(^**^Q^**^)^ (Bolger et al., 2014), AmpliconNoise^(^**^Q, M^**^)^ (Bragg et al., 2012), UNOISE^(^**^M^**^,^ **^U^**^)^ (Edgar, 2016), UCHIME^(^**^Q^**^,^ **^M^**^,^ **^U^**^)^ (Edgar et al., 2011), Deblur^(^**^Q^**^,^ **^M^**^)^ (Amir et al., 2017), and DADA2^(^**^Q^**^)^ (Callahan et al., 2016)
	Remove host DNA contaminant reads	Bowtie2^(^**^Q^**^)^ (Langmead and Salzberg, 2012), BMTagger (Agarwala and Morgulis, 2011), and DeconSeq (Schmieder and Edwards, 2011)
(2) Sequence assembly	*De novo* read assembly	MEGAHIT (Li et al., 2015), MAFFT^(^**^Q^**^,^ **^M^**^)^ (Katoh and Standley, 2013), UCLUST^(^**^Q^**^,^ **^U^**^)^ (Edgar, 2010), and metaSPAdes^(^**^Q^**^,^ **^M^**^)^ (Nurk et al., 2017)
	Read alignment to annotated database	DIAMOND (Buchfink et al., 2014), NAST^(^**^Q^**^,^ **^M^**^)^ (DeSantis et al., 2006), USEARCH^(^**^Q^**^,^ **^U^**^)^ (Edgar, 2010), and VSEARCH^(^**^Q^**^,^ **^M^**^)^ (Rognes et al., 2016)
(3) OTU analysis	Assignment of reads to OTUs	UPARSE-OTU^(^**^U^**^)^ (Edgar, 2013), Kraken (Wood and Salzberg, 2014), MetaPhlAn2^(^**^Q^**^)^ (Truong et al., 2015), and DOTUR^(^**^M^**^)^ (Schloss and Handelsman, 2005)
(4) Functional profiling	Functional profiling and prediction	MEGAN (Huson et al., 2016), HUMAnN (Abubucker et al., 2012), MetaCLADE, MOCAT (Kultima et al., 2016), and PICRUSt (Langille et al., 2013)
(5) Diversity analysis	Diversity, evenness, and richness metrics	Alpha [e.g., Chao1^(^**^Q, M, U^**^)^] and Beta [e.g., Jaccard^(^**^Q, M, U^**^)^]

#### Challenges in Microbiome Data Processing

Growth in the variety and complexity of data processing tools presents opportunities but also significant challenges for new investigators. First, although best practices have been suggested (Knight et al., 2018), tools are still far from a fully automated user experience that would lead to reliable results. Second, microbial genomes with different abundances are sequenced together, making metagenomic assembly more challenging compared to single genome assembly where the sequence coverage is approximately uniform. Third, the number of uncharacterized microbes (known as microbial dark matter) exacerbates problems associated with unaligned and misaligned sequence reads. Fourth, evaluation of methodology and findings from different studies is difficult since each study may use a different method or a different implementation of the same method in their data processing pipeline. Fifth, data collection and integration of microbiome data from different studies are difficult because of many factors including differences in wet-lab library preparation (e.g., primers used), differences in sequencing devices and their settings (e.g., coverage), and non-uniform methods of formatting and storage for microbiome data and metadata. See Quince et al. (2017) for further discussion concerning microbiome data processing challenges.

### Data Analytics and Machine Learning

Data processing is considered to be the step necessary for converting the raw data, such as metagenomics sequence reads, into biologically meaningful representations, such as OTU counts using bioinformatics tools, some of which are done in the sequencing device itself. Data analytics, start after the integration of processed sample data from various information sources (i.e., microbiota, diet, and host), as illustrated in Figure 3. In most cases, all samples are from a single study, which helps ensure consistency with respect to the experimental settings and data processing protocols used. Furthermore, limited resources force the researchers to narrow their data collection to particular information types in order to have sufficient statistical power for hypothesis testing. A recent increase in the number of microbiome studies with publicly available data has enabled cross-study data integration (Pasolli et al., 2016, 2017; Duvallet et al., 2017; Wang et al., 2018; Thomas et al., 2019; Wirbel et al., 2019). In such cases, extra precautions are necessary to minimize biases introduced by inconsistencies among datasets during data collection, sample preparation, sequencing, and data processing.

#### Challenges in Microbiome Data Analysis

A number of challenges arise when analyzing microbiome data, as summarized in Table 3. The first challenge is due to *compositional quantities* in microbiome data. Quantities such as the number of reads assigned to a given species, which can only be interpreted as proportions, are called compositional. These quantities cannot be compared directly across multiple samples. Conclusions should not be made based on the number of reads assigned to individual sample features (e.g., OTUs, genes, and functional groups) since they do not represent absolute abundances due to instrumental limitations (Gloor et al., 2017). Instead, the assigned number of reads should be converted to relative abundances and analyzed with that in mind. Some studies perform rarefaction to adjust for differences in library size due to unexhaustive metagenomic sampling. Although several pipelines provide this functionality, it has been found inadmissible for metagenomics microbiome studies as it discards many reads leading to decreased sensitivity in differential abundance testing (McMurdie and Holmes, 2014) and biased estimates for alpha diversity (Willis, 2019). The second challenge is due to the *high dimensionality* associated with OMICS data. Datasets in which items are characterized by a high number of features while the number of items is limited are called high dimensional. In microbiome studies, a limited number of individuals are characterized using many host, diet, and microbiome features leading to high dimensional datasets (Li, 2015). Dimensionality can be reduced by grouping OTUs into phylogenetic variables, regularization, or unsupervised dimensionality reduction (DR) (explained below). The third challenge is about testing *multiple hypotheses* in an exploratory analysis. It relates to the fact that, as the number of hypotheses increases, the chance of false discoveries also increases. This can be addressed by increasing sample size and *p*-value adjustment (explained below). The fourth challenge relates to *hierarchical relationships* amongst bacterial species due to their shared ancestors. Assumptions such as independence among samples may not hold, leading to wrong estimations of correlation (Felsenstein, 1985) and phylogeny-aware methods to address the issue. The fifth challenge is about *missing quantities* in sampled data. For example, when marker gene sequencing is used, quantities relating to the amounts of functional genes in the microbiome are not directly available (i.e., missing). Identifying functions of microbial organisms is important for understanding the gut microbiota. Such information can be estimated using metatranscriptomics data, which is often not available. Data imputation tools, such as PICRUSt (Langille et al., 2013), help to mitigate this through gene imputation based on annotated databases.

**TABLE 3:** Key challenges that arise in microbiome data analysis with examples and suggested solutions.

Challenges in microbiome data analysis	Examples and solutions
**(1) Compositional quantities:** Metagenomic data processing provides read counts for discovered entities such as genes, species, and OTUs from a given sample. These read counts are only meaningful within a sample.	**Example:** Metagenomic analysis of feces samples tells us that Person A has 5 reads mapped to bacterium *Escherichia coli*, while person B has 10. Can we conclude that this bacterium is more populated in the gut of person B compared to person A? *Answer*: No, read counts cannot be compared across samples.
	**Solutions:** (I) Convert read counts to relative abundances before comparison. (II) If an optimization problem is defined using read counts, add constraint for total counts per sample.
**(2) High dimensionality:** Metagenomic data processing results in many entities such as genes and species discovered for each sample, which may not be shared among multiple samples. During data aggregation, one dimension is associated to each entity resulting in a high number of dimensions compared to the number of samples.	**Example:** Metagenomic data processing of feces samples from 20 individuals results in relative abundances for 10 microbial families per sample. Can we use classical linear regression to predict an individual’s age using relative abundances from aggregated data? *Answer:* No, aggregating 20 samples results in more than 20 microbial families.
	**Solutions:** (I) Use dimensionality reduction such as PCA prior to regression. (II) Use regularized linear regression such as Lasso. (III) Use microbial abundances of higher-order taxonomic ranks such as phylum instead of family.
**(3) Multiple hypotheses:** The high-dimensional nature of metagenomic data allows the researcher to generate a large number of hypotheses, which leads to seeing patterns that simply occur due to random chance. This is sometimes called “the high probability of low probability events.”	**Example:** Metagenomic data processing provides relative microbial abundances at species level using feces samples of 200 individuals, half of which are diagnosed with Crohn’s disease and the rest are healthy. Performing a *t*-test identifies that the relative abundance of 40 species (amongst 1,000) are significantly different between microbiota of sick and healthy individuals (*p-value* < 0.05). Is this result correct? *Answer:* No, the standard threshold of 0.05 for *p-value* is only acceptable when a single hypothesis is involved while the *t*-test is performed 1,000 times leading to many false discoveries.
	**Solution:** Calculate FDR adjusted *p-value* (i.e., *q-value*) of 0.05 to control the false discovery rate.
**(4) Hierarchical relationships:** Assumptions of independence do not hold in microbiome data since taxonomic variables (e.g., species and OTUs) have known hierarchical relationships due to genetic and phenotypic similarities. Therefore, common statistical techniques that assume independence between variables are problematic.	**Example:** Beta-diversity can be used to calculate the similarity between groups of microbiome samples. Can we simply calculate the Beta-diversity using standard Euclidean distance between relative abundances at a given taxonomic order? *Answer:* No, Euclidean distance doesn’t take into account the similarity between species.
	**Solution:** Use phylogeny-aware metrics such as UniFrac distance instead, which takes into account the phylogenetic tree when calculating distances.
**(5) Missing quantities:** Metagenomic data often lacks information about the functions of the microbial communities which can only be estimated using meta-transcriptomics or meta-proteomics. However, deciphering microbiota’s function is a major goal in microbiome studies.	**Example:** In one case, metagenomic data processing from marker-gene data has provided us with relative abundances at the genus level, but we do not know the possible functions of the microbiota in terms of proteins that it can produce. Should we abandon further analysis? *Answer:* No, although we don’t have direct information about proteins, we can infer.
	**Solution:** Databases such as Greengenes contain the whole-genome sequence of identified species at various taxonomic orders which can be used for gene and protein inference.

The methods for identifying microbiota characteristics associated with host phenotypes of interest can be categorized into two main groups, based on whether they use supervised or unsupervised learning. Supervised learning methods require labeled data, while unsupervised learning methods can be used when records are not labeled. More advanced methods include semi-supervised learning (Zhu, 2005), which takes advantage of both labeled and unlabeled data, and transfer learning (Pan and Yang, 2010), which transfers knowledge learned from one task to another, are not discussed here.

#### Supervised Learning Methods

##### Hypothesis testing and variation analysis

Analysis of variation may involve single or multiple variables. For a single variable hypothesis, the student’s *t*-test or non-parametric tests, such as Wilcoxon rank-sum or Kruskal–Wallis, can be used. For example, the *t*-test has been used to show that patients with ADHD have a lower alpha-diversity index of gut microbiota compared to healthy controls (Prehn-Kristensen et al., 2018). Non-parametric tests are good alternatives when the assumptions regarding the data being normally distributed do not hold. For example, the Wilcoxon rank-sum test is used on predicted pathway data, suggesting that enzymes in the “Glycan Biosynthesis and Degradation” pathway increase in summer when compared to winter (Davenport et al., 2014). In cases where a statistical test is repeated with different hypotheses (i.e., multiple hypothesis testing), the statistical significance should be adjusted by methods such as an FDR adjustment (i.e., *q*-value) (Benjamini and Hochberg, 1995) or Holm’s procedure (Rice, 1989).

When the hypothesis that is investigated contains multiple variables, MANOVA (Smith et al., 1962) or non-parametric alternatives such as PERMANOVA (Anderson, 2001) or ANOSIM (Clarke, 1993) can be used. The samples are first assigned to multiple groups (e.g., based on some feature values). The goal is to quantify how much this grouping can explain the distribution of values in any given sample feature (response variable). The simplest case is the popular method called analysis of variance (ANOVA), which considers a single response variable with a normal distribution. For instance, in a recent study, two bacterial phyla (Bacteroidetes and Firmicutes) were identified using ANOVA with different relative abundance in the microbiota of children living in a rural African village compared to European children (De Filippo et al., 2010). ANOVA can be generalized to multivariate analysis of variance (MANOVA), which can have multiple response variables. For example, it is used to investigate the overall difference in composition between the microbiota of children with Prader–Willi syndrome and children with simple obesity, before and after treatment (Zhang et al., 2015). In many cases, normal distribution assumptions do not hold; hence, non-parametric methods are used. In one study, PERMANOVA is used to detect taxonomic differences in the microbiota of patients with Crohn’s disease when compared to healthy controls (Pascal et al., 2017).

##### Regression and correlation analysis

A general understanding of the extent of association among pairs of variables can be achieved using correlation analysis. Correlation metrics measure different types of relationships. For example, the Bray–Curtis measures abundance similarities (Bray and Curtis, 1957), the Pearson correlation coefficient quantifies linear relationships, and the Spearman correlation coefficient quantifies rank relationships (Spearman, 1904). In (Weiss et al., 2016), the authors perform a simulation-based comparison on a range of correlation metrics for microbiome data. Metrics such as SparCC (Friedman and Alm, 2012) and LSA (Ruan et al., 2006) perform particularly better as they are designed to capture complex relationships in compositional microbiome data. For example, SparCC is used for analyzing the TwinUK dataset to identify bacterial taxa whose abundances are influenced by host genetics (Goodrich et al., 2014). This was done by creating a correlation network between microbial families based on their intraclass correlation. More recently, the phylogenetic isometric log-ratio (PhILR) transform has been introduced (Silverman et al., 2017) to transform compositional data into non-compositional space where standard data analytic techniques are applicable. Usage of such transformations should be limited to features that are compositional and phylogenetic in nature.

Regression methods aim to predict the change in one continuous variable using other variables. Correlation analysis can be considered a special case of regression with a single input variable. Standard linear regression can be used for various DGMH predictive tasks. However, when variables relate to OTU abundances, the typical assumptions of a linear relationship, normal distribution, and dependence do not hold. For example, when the goal is to predict the composition of OTUs [normalized for summing up to one (Tyler et al., 2014)], zero-inflated continuous distributions are used. Often a two-part regression model is used where part I is a logistical model to calculate the probability that the given OTU is present. Part II is a generalized linear regression using beta distribution to predict relative abundance assuming the presence of OTU in the sample (Ospina and Ferrari, 2012; Chen and Li, 2016; Peng et al., 2016). Phylogenetic comparative methods (PCMs) such as phylogenetic generalized least squares (PGLS) are used to control for dependence among observations given the phylogenetic hierarchies (Washburne et al., 2018). Ignoring the phylogenetic ancestry of microbial species can increase the chance of false discovery in regression models (Felsenstein, 1985). PCMs are not widely used in microbiome studies today, which may be one reason for a high number of false positives that can be alleviated by using them (Bradley et al., 2018).

Canonical correlation analysis (CCA) can be used (Hotelling, 1992) to investigate the correlation between two groups of variables (e.g., abundances of microbiome OTUs and metabolites). CCA finds linear transformation pairs that are maximally correlated when applied to data while ensuring orthogonality for different transformation pairs. The original CCA, however, fails for high dimensional microbiome data when the number of variables exceeds the number of samples. This can be addressed using regularization, giving rise to sparse CCA methods (Witten et al., 2009). For example, a sparse CCA is applied to investigate correlations between the gut microbiome and metabolome features in type 1 diabetes (Kostic et al., 2015).

##### Classification

In supervised classification, the goal is to build a predictive model (classifier) using labeled training data. The labels can have binary or categorical values (in contrast to regression where labels are continuous and numerical). In one study, a classifier was built to predict the geographical origin of sample donors using relative OTU abundances estimated from 16s rRNA gut samples (Yatsunenko et al., 2012). This was done using the method called Random Forests (RF), which constructs an ensemble of decision trees (Breiman, 2001). In a different study, the classification task was to identify healthy vs. unhealthy donors given relative OTU abundance data (including species level) coming from shotgun metagenomics sequencing of the gut (as well as other body sites) (Pasolli et al., 2016). In addition to RF, they used the support vector machine (SVM) classifier, which is a powerful method for building generalizable and interpretable models and is mathematically well understood (Suykens and Vandewalle, 1999). In their study, RF classifiers performed better than SVM except in a few datasets. Both RF and SVM have built-in capability to deal with overfitting issues that arise in high-dimensional datasets. RF achieves this using an ensemble-based technique where the prediction is made based on predictions from many trained classifiers. In SVM, parameters of the predictive model are constrained based on *a priori* defined criteria. Note that constraining the model parameters is often mathematically equivalent to regularization (Scholkopf and Smola, 2001). In both cases, the objective is to minimize the value of a loss function that calculates the overall error in model predictions. When regularization is used, the loss function not only depends on prediction errors but also on the magnitude of model parameters. For example, in L1 regularization, the absolute values of model parameters are scaled and added to the loss function. Therefore, when two models have a similar error, the model with smaller parameter values will be selected during training. L1 regularization is commonly used for feature selection by picking only the non-zero features of the trained model because such a model achieves a low prediction error while using a subset of features.

Artificial neural networks (ANN) can also be used for classification and are shown to outperform other techniques in many areas of biology (Kim et al., 2016, 2017; Singh et al., 2016; Eetemadi and Tagkopoulos, 2018) as well as computer vision and natural language processing, to name a few (LeCun et al., 2015). Recently, a new ANN-based method called Regularization of Learning Networks (RLN) was designed and evaluated microbiome data. RLN provides an efficient way for tuning regularization parameters of a neural network when a different regularization coefficient is assigned for each parameter (Shavitt and Segal, 2018). They use RLN to predict human traits (e.g., BMI, cholesterol) from estimated relative OTU abundances and gene abundances. We expect the development of new classification methods that can deal with the aforementioned challenges arising in DGMH data by considering the biological phenomenon, properties of measurement instruments, and upstream data processing pipelines.

#### Unsupervised Learning Methods

##### Dimensionality reduction

High-dimensional datasets can provide a high resolution and multifaceted view of a phenomenon such as gut microbiota. Predictive performance in data analytics can increase significantly given such data. Many data analytics methods, however, fall short when presented with high-dimensional data that necessitates using DR. Once dimensionality is reduced, data visualization and analytics become more accessible. Principal component analysis (PCA) is one of the most widely used DR methods. It replaces the original features with a few uncorrelated features called principal components (PCs), which are linear combinations of the original features and preserve most of the variance within the data. In one study, PCA was applied to predicted abundances of about 10 million genes from the gut microbiota of donors (Li et al., 2014). Reducing dimensionality from 10 million to two dimensions only enabled visualization of data on a standard two-dimensional scatter-plot (i.e., PCA plot) showing a clear distinction between the microbiota of Danish and Chinese donors. In another study, the top five PCs of individual bacteria’s genome (sequenced from infant fecal samples) were used to create a classifier for predicting antibiotic resistance (Rahman et al., 2018).

The relationships among features in a microbiome study can be used in DR, giving rise to various factor analysis (FA) methods we review here briefly. Multiple factor analysis (MFA) is an extension of PCA that considers predefined grouping of features during DR to ensure equal representation for all groups of features in derived PCs (Abdi et al., 2013). In one study (Robertson et al., 2018), MFA is used for simultaneous 2D visualization of host and microbiome features (see Morgan et al., 2012; Raymond et al., 2016 for other examples). Exploratory factor analysis (EFA) is used to identify unobserved latent features called factors to explain the correlations among observed features (Yong and Pearce, 2013). Factors that are identified by EFA are uncorrelated to each other similar to PCs in PCA; however, PCs are used to explain overall variance instead of correlations. EFA has been used in a recent study to extract four factors explaining the correlations among 25 top taxa for studying the association of microbiome with early childhood neurodevelopmental outcomes in 309 infants (Sordillo et al., 2019). Confirmatory factor analysis (CFA) and structural equation modeling (SEM) can be used to examine the extent to which a hypothesized model of latent features and their relationships with observed variables are supported by the data (Schreiber et al., 2006). In a recent study, a theoretical framework is proposed and examined using CFA to model the influence of maternal and infant factors on the milk microbiota (Moossavi et al., 2019). The R packages lavaan (Rosseel, 2012) and FactoMineR (Lê et al., 2008), as well as the IBM SPSS software (IBM Corp, 2013), are widely used for factor analysis.

Another related method is principal coordinate analysis (PCoA), also called multidimensional scaling (MDS) (Kruskal, 1964), which is commonly employed for 2- and 3-dimensional visualization of beta diversity. It can deal with situations where distances between individual feature vectors from samples cannot be used directly (e.g., due to significant sparsity and phylogenetic relationships). PCoA takes a matrix of distances among samples (e.g., UniFrac distance between OTU abundances of a pair of sample donors) as input. It then assigns new coordinates such as PC1 and PC2 to each sample such that the Euclidean distances in the new coordinate are similar to the ones in the matrix. For example, PCoA was applied given UniFrac distances between OTU abundances (from 16S rRNA samples) from the gut microbiota of donors (Yatsunenko et al., 2012). Two-dimensional visualization using PC1 and PC2 showed that the gut microbiota of donors who lived in the United States is distinct from the gut microbiota in donors living in Amerindian and Malawian villages.

Linear discriminant analysis (LDA) is also a DR technique, although supervised and closely related to regression and ANOVA. Unlike PCA and PCoA, it requires class labels. It generates new features that are linear combinations of the original ones while separating samples with respect to their class labels. In one study, LDA was used to distinguish gut microbiota samples based on diet but not for DR (Paulson et al., 2013). Successful usage of LDA for high dimensional microbiome data may require regularization to account for overfitting as similarly used for high-dimensional microarray (Guo et al., 2006).

The optimal amount of reduction in dimensionality (e.g., the number of principal components) varies given the data and the task downstream. For data visualization tasks, it is largely constrained by the limitations of human visual perception (three dimensional). For downstream supervised learning tasks, we are often interested in the maximum amount of DR without a significant decrease in predictive power. This is showcased in Bartenhagen et al. (2010), where the impact of the amount of DR on classification performance is evaluated for gene expression data.

##### Cluster analysis

Similar microbial communities are expected to exhibit analogous effects on the host organism (Gould et al., 2018). Once a similarity measure is defined, various cluster analysis methods can be used to find groups of samples with similar microbiota. In one study, three robust microbiota clusters (called enterotypes) were identified using cluster analysis from 16s rRNA data of fecal samples (Arumugam et al., 2011). It was later shown that such clustering results are not only sensitive to data but also to choices made during analysis (Koren et al., 2013). We enumerate four important choices impacting cluster analysis results (other than upstream data processing). First is the distance measure. Standard distance metrics such as the Euclidean and Manhattan distance are simple, well understood, and supported in many clustering libraries. Applicability of such metrics depends on prior compositionality aware transformations such as ILR. Beta-diversity metrics such as weighted and unweighted UniFrac distances are designed for microbiome analysis considering compositionality and phylogenetic dependencies of microbiome data. Researchers should pay attention to the properties of the distance metric used in order to better understand the clustering results. Second is the clustering algorithm. Algorithms such as Partition Around Medoids (Kaufman and Rousseeuw, 1987) and Hierarchical Clustering (Murtagh and Contreras, 2012) can employ various distance metrics. Others, such as *k*-means, are tied to a single distance measure but computationally less demanding. Third is the number of clusters. Clustering algorithms often require the number of clusters to be provided as input. When unknown, the number that provides higher cluster scoring is picked. Prediction strength (Tibshirani and Walther, 2005), silhouette index (Rousseeuw, 1987), and Calinski–Harabasz (Caliñski and Harabasz, 1974) are popular cluster scoring metrics. Fourth is the method used to identify the robustness of clustering results. Often a cluster scoring metric that is not used to identify the number of clusters is used as a robustness measure. Recent studies consider the effect of the above choices during cluster analysis to better understand how results can be generalized (Hildebrand et al., 2013; Costea et al., 2018).

The integration of data from disparate omics data types (also called integrative omics) and other heterogeneous metadata enables a more comprehensive look into the underlying biology (Karczewski and Snyder, 2018). Integrative omics data analysis methods have been categorized into three types (Kim and Tagkopoulos, 2018). First is *data-to-data*, where disparate data types are analyzed together. For example, CCA can be used to investigate the correlations between metagenomics and metabolomics data, as discussed before. Second is *data-to-knowledge*, where the knowledge gained from analyzing some data types are used to inform analysis of other data types. For example, a metagenomics analysis of colon cancer patients can lead to further investigation of candidate genes using targeted proteomics analysis. Third is *knowledge-to-knowledge*, where the data types are initially analyzed separately, but the acquired knowledge is integrated together afterward to either identify hypotheses that are supported by multiple data types or create a more complete view of a given phenomenon. For example, differentially expressed genes and differentially abundant metabolites in the digestive tract of patients with Crohn’s disease can be used together for narrowing down pathways involved in disease etiology. See Huang et al. (2017), Karczewski and Snyder (2018), Kim and Tagkopoulos (2018), and Jiang et al. (2019) for comprehensive reviews.

### Recommendation Systems and Artificial Intelligence

The human microbiome is referred to as “our second genome” and has a major influence on our health (Grice and Segre, 2012). Although it is known for its resilience (Lozupone et al., 2012; Relman, 2012), unlike the human genome, it has considerable plasticity hence providing ample opportunities in the design of new types of food, medical interventions, and dietary recommendations (Gentile and Weir, 2018). Despite recent progress in microbiome research, switching from population-wide dietary recommendations to microbiome-aware recommendations is not yet realized. See Table 4, for a representative summary of recent microbiome-aware diet recommendation studies. Once a personalized healthy target microbiome is identified using data analytics methods, a recommendation system (RS) can utilize this information to suggest the path toward establishing it in the host and ensuring the health benefits. One approach is to use a knowledge-based RS where recommendations are made using a limited number of approved drugs and dietary prescriptions. Although this would be a good starting point, such a system would be limited in its ability to provide precise and personalized recommendations that usually need a platform that can create new products or processes on a case-by-case basis. Recent studies simulate a virtual gut microbiome by integrating known metabolic pathways of microbial species with the individual’s microbiome and diet (Shoaie et al., 2015; Baldini et al., 2018; Bauer and Thiele, 2018; Greenhalgh et al., 2018). Such mechanistic modeling is very promising, however, it is currently hindered by numerous challenges, such as incomplete characterization of an individual’s gut and metabolic pathways of their microbiome. There is considerable research on AI-based RS related to food, drug design, and health (Tran et al., 2017; Suphavilai et al., 2018), but its application with microbiome data is in its early stages (Zeevi et al., 2015; Thaiss et al., 2016a; Korem et al., 2017). Commercial investments in this area have already started, with companies such as UBiome and DayTwo using 16S rRNA technology to provide insights into our personal microbiota and suggest dietary recommendations.

**TABLE 4:** Highlighted microbiome-aware diet recommendation studies.

Study description	Dietary variables	Metagenomic technology	References
A personalized meal recommendation system uses personal, microbiome and dietary features to select an optimal meal for lowering post-meal glucose levels in patients with type II diabetes.	Micro and macronutrients	16S rRNA and whole metagenomics	Zeevi et al., 2015
Microbiome features enable accurate prediction of an individual’s glycemic response to different bread types.	Bread type	16S rRNA and whole metagenomics	Korem et al., 2017
Accurate prediction of weight regain given normal vs. high-fat diet in mice is enabled using a microbiome-based predictor.	Dietary fat	16S rRNA	Thaiss et al., 2016a
Personalized metabolite supplement recommendations for Crohn’s disease are made using *in silico* simulation of reconstructed metabolic pathways from gut microbiome (773 microbes).	Metabolic supplements	Whole metagenomics	Bauer and Thiele, 2018
Fecal amino acid levels are predicted given dietary macronutrients through *in silico* simulation of metabolic pathways from gut microbiome (four microbes) and host cells.	Macronutrients	16S rRNA	Shoaie et al., 2015
			

Recommendation system is defined as “any system that guides a user in a personalized way to interesting or useful objects in a large space of possible options or that produces such objects as output” (Burke, 2002). Microbiome-aware diet recommendations can be generated from knowledge-based, content-based, or collaborative filtering, as described next.

#### Knowledge-Based Recommendation Systems

An ideal knowledge-based RS would be based on *in silico* models that can correctly simulate an individual’s gut. It requires proper characterization of the gut microbiome, human intestinal cells, intestinal and dietary metabolite concentrations, their interactions through metabolic pathways, and realistic objective functions for modeling such complex dynamics. Such a knowledge-based RS was devised in a recent study involving 28 patients with Crohn’s disease and 26 healthy individuals (Bauer and Thiele, 2018). Researchers integrated genome-scale metabolomic reconstructions (GENREs) of 818 microbes from http://vmh.life (Noronha et al., 2018) with the individual’s microbiome abundances after metagenomic data processing in the R package BacArena (Bauer et al., 2017). Their *in silico* simulations provide personalized metabolic supplements for improving patient’s SCFA levels. Earlier studies have created a metabolic model of the gut microbiome on a smaller scale (Shoaie et al., 2015). See Magnúsdóttir and Thiele (2018) for a comprehensive review. Despite their promise, there are several challenges for the application of such knowledge-based RSs. The first challenge is the limited availability and accuracy of GENREs for gut microbes. A recent study has identified 1,520 unique microbes in the human gut (Zou et al., 2019), while the number of microbes that have GENREs is only 818 (Noronha et al., 2018). In one study (Tramontano et al., 2018), 75% of the GENREs required updates [from previously constructed GENREs (Magnúsdóttir et al., 2017)] so that *in silico* simulations could recapitulate growth on new media. This suggests that *in silico* GENREs of the gut microbiome are far from complete, however, progress is being made toward closing this gap. The second challenge is the metabolic characterization of the media inside the intestine on which gut microbes grow. This includes identifying the dietary metabolites available to microbes at different sites in the gut, which necessitates meticulous dietary data processing. The third challenge relates to the computational complexity of *in silico* simulations, which increases as host and microbial GENREs become more comprehensive. Although more challenges can be enumerated, their inclusion here would go beyond the scope of this article.

#### Content-Based Recommendation Systems

In content-based RSs, the recommendations are made based on the item’s content (often characterized using item features). This is in contrast to collaborative filtering RSs where recommendations are based on preferences of other users for each item. In one landmark study (Zeevi et al., 2015), authors use a content-based RS for meal recommendations with the goal of improving post-meal glucose levels. Each meal is first characterized based on its nutritional profile (macronutrients and micronutrients). Then a regression model is trained to predict post-meal glucose level based on the meal’s nutritional profile, the individual’s microbiome features, and other personal information. For each new user and meal, post-meal glucose levels are predicted by the model, and the meal with the minimum post-meal glucose level is recommended to the user. The same methodology is used in a later study using only microbiome features of individuals to predict post-meal glucose levels in a bread-type recommendation system (Korem et al., 2017). Several challenges arise when building content-based RSs. The first challenge is variable data quality and compatibility. When a group of users (or items) are overrepresented in the data, the predictive model tends to be biased toward their favorite items. As a result, the quality of recommendations will be highly variable. Stratified sampling can be used to alleviate this issue. The second challenge is difficulty in generalizing and personalizing recommendations, particularly when feature vectors are not informative for predictions (also relevant to the “missing quantities” challenge mentioned in Table 3). This is in contrast to collaborative filtering RSs, where latent features are learned instead of being defined *a priori*. Hybrid RS methods are designed to take advantage of collaborative filtering RSs to address such inherent challenges in context-based RSs (and vice versa) (Burke, 2002). For an extensive review of context-based RSs, methods see (Lops et al., 2011).

#### Collaborative Filtering Recommendation Systems

In collaborative filtering RSs, each user is characterized by the items (foods or ingredients here) they have previously rated, bought, or generally acted upon. Recommendations are given based on the idea that users who assign the same rating to existing items are expected to have a similar rating profile for all items. Matrix completion is one of the most popular collaborative filtering methods (Su and Khoshgoftaar, 2009; Ekstrand et al., 2011). User-assigned scores are first organized in a sparse matrix where columns correspond to different items and rows to various users. In cases where most users only have evaluated a few items, most of the matrix remains empty. Matrix completion fills the rest of the matrix through the similarities discovered amongst users and items. See Su and Khoshgoftaar (2009) and Ekstrand et al. (2011) for a comprehensive review. Collaborative filtering RSs have not been used for microbiome-aware food recommendations. We describe an example here to showcase how it can be used. Consider a matrix where each column corresponds to a dietary plan and each row to a person—a specific value can represent gut microbiome alpha diversity during the time which the user followed a particular dietary plan. Assuming that each person has only tried a few dietary plans, most of the matrix will be empty. Here we can use matrix completion to fill the matrix with predicted alpha diversities to create a complete matrix. This can be used to recommend dietary plans for a person with the goal of maximizing gut microbiota diversity. Several challenges arise in collaborative filtering RS. The first challenge is the lack of data for new users (“cold-start”). Note that the recommendations rely on similarities among users, while new users have not tried any of the items available in the database. The second challenge is the curse of dimensionality. As the number of items increases, the chance of having user scores for the same item combinations decreases, hence items and users become equally dissimilar (also relevant to the “high dimensionality” challenges in Table 3). In such cases, hybrid RS can be used. Next, we bring up a few example scenarios.

### Example Scenarios

**FIGURE 4:**
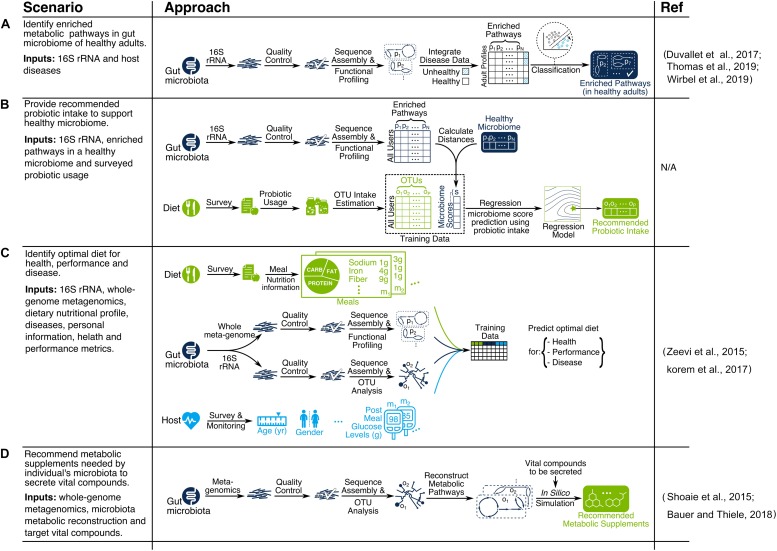
Examples of microbiome-aware diet recommendation pipelines for scenarios **(A–D)**.

We discussed various data analytics and recommendation system methods for microbiome discovery and diet engineering, as illustrated in Figures 1, 3. Applicability of each method depends on research objectives and data availability. Here we explain particular scenarios illustrated in Figure 4 as blueprints for integrating relevant techniques in a single pipeline. In scenario A, the goal is to identify metabolic pathways that are enriched in the gut microbiome of healthy adults using 16S rRNA data (see Duvallet et al., 2017; Thomas et al., 2019; Wirbel et al., 2019 for similar works). In scenario B, the goal is to provide recommended probiotic intake for supporting a healthy gut microbiome. First, the study participants would be profiled based on the probiotic products they consume (each containing specific OTUs) as well as their gut microbiome. Next, microbiome scores will be calculated for each participant based on the distance between enriched pathways of their microbiome and the target healthy microbiome. Then a regression model is trained to predict microbiome scores based on OTU intakes. Finally, the OTU intake concentration that is predicted to have an optimal microbiome score would be used as the recommended probiotic intake. In scenario C, the goal is to identify optimal diets for health, performance, and disease. A compendium needs to be built following a consistent data collection and processing pipeline for study participants. The compendium serves the training data necessary for building machine learning models to predict health metrics such as post-meal glucose level (Zeevi et al., 2015; Korem et al., 2017) or post-dieting weight regain (Thaiss et al., 2016a). The predictive models can then be used as the key part of a recommendation system by identifying the expected impact of a given diet on health for new individuals. In scenario D, the goal is to recommend metabolic supplements needed by an individual’s microbiota to secrete vital compounds. First, OTU abundances of each individual are identified using a metagenomic data processing pipeline. Then, individual gut metabolic pathways are reconstructed using online resources such as the Virtual Metabolic Human database (Noronha et al., 2018). Finally, constraint-based reconstruction and analysis (COBRA) tools (Bauer et al., 2017; Baldini et al., 2018) are used to perform *in silico* simulations of GENREs to identify metabolic intake requirements to secrete vital compounds of interest. This mechanistically sound approach has been used in a few recent studies (Shoaie et al., 2015; Bauer and Thiele, 2018).

## Intellectual Property Development

The potential application impact generated by research on the relationship between the gut microbiome and diet can be visualized by the abundant number of patent applications on the topic, as well as more generally in the field of microbiome and health research. A search for “gut microbiome” and “diet” returns over 2,500 patents on Google, deposited by universities, institutes, and companies such as MicroBiome, Microbiome Therapeutics, Gutguide, Whole Biome Inc., UBiome, and others, from as early as 2004. However, it is important to note that most of these hits are less than a decade old, demonstrating the relatively early stages in which this area still resides. The exponential growth in patent applications related to the microbiome since 2007 correlates to a similar curve for the academic publications in the same period (Fankhauser et al., 2018).

One of the earliest available patent applications (US20050239706A1) related to the topic of the microbiome and nutrition describes methods to regulate weight by manipulating the gut microbiome. Additional patents also aim to use the gut microbiome as a therapeutic target, monitoring and altering the composition with the goal of manipulating the host phenotype such as weight gain/loss and obesity. In general, weight management with the manipulation of the gut microbiome (US20110123501A1 and US20100172874A1) appears as a favored theme for early patent applications in the area of microbiome and diet. Several patents describe novel probiotics and their uses (WO2007136553A2), often relating them to specific target phenotypes such as weight loss (EP2178543B1, US9371510B2, US9113641B2, EP2216036A1, EP2296489A1, and WO2010091991A1). Multiple applications for probiotics focused on weight loss were deposited by Nestec SA, which offers research and consulting services to the food company Nestlé S.A.

With the development of computational techniques to analyze larger datasets, and more research on the relationship of the microbiome and the host homeostasis and disease, patent applications related to gut microbiome and diet have subsequently extended to other health conditions beyond obesity and weight control. Among the newest patent applications related to the gut microbiome and diet is a patent describing the characterization, diagnostics, and treatment of a locomotor system condition based on microbiome data (US20170372027A1). Other applications include metagenomic methods specific for the comparison of healthy individuals and those with gut dysbiosis (WO2017216820A1), diagnostic tools for Crohn’s disease, inflammatory bowel disease, irritable bowel syndrome, ulcerative colitis, and celiac disease using microbiome and other types of data (US20170286620A1), and devices such as capsules to acquire and monitor microbiome and metabolites in the gut (US20170281091A1). Research on the gut–brain axis relationship also resulted in several applications aimed at monitoring and manipulating the gut microbiome to enhance cognition or treat mental-health conditions (WO2017171563A1 and WO2017160711A1). A recent and thorough review of patents related to the microbiome identified cancer diagnosis and treatment and CRISPR technology as recent trends in the field (Fankhauser et al., 2018). Table 5 shows a summary of highlighted patents relating to DGMH.

**TABLE 5:** Highlighted patents relating to diet, gut microbiome, and human health.

Patent number	Name	Owner	Year
US20100172874A1	Gut microbiome as a biomarker and therapeutic target for treating obesity or an obesity-related disorder	Washington University in St. Louis	06
WO2007136553A2	Bacterial strains, compositions including same and probiotic use thereof	Benson et al.	06
US20110123501A1	Gut flora and weight management	Nestec S.A.	07
EP2178543B1	*Lactobacillus rhamnosus* and weight control	Nestec S.A.	07
US9371510B2	Probiotic compositions and methods for inducing and supporting weight loss	Brenda E. Moore	07
US9113641B2	Probiotic bacteria and regulation of fat storage	Arla Foods amba	07
EP2296489A1	*Lactobacillus paracasei* and weight control	Nestec S.A.	08
EP2216036A1	*Lactobacillus rhamnosus* NCC4007, a probiotic mixture and weight control	Nestec S.A.	09
WO2010091991A1	*Lactobacillus helveticus* cncm i-4095 and weight control	Arigoni et al.	09
US20100331641A1	Devices for continual monitoring and introduction of gastrointestinal microbes	Gearbox LLC	09
US20160074505A1	Method and System for Targeting the Microbiome to Promote Health and Treat Allergic and Inflammatory Diseases	Kovarik et al.	09
US20120058094A1	Compositions and methods for treating obesity and related disorders by characterizing and restoring mammalian bacterial microbiota	New York University Dow Global Technologies LLC	10
US9040101B2	Method to treat diabetes utilizing a gastrointestinal microbiome modulating composition	MicroBiome Therapeutics LLC	11
US20170348359A1	Method and System for Treating Cancer and Other Age-Related Diseases by Extending the Health span of a Human	Kovarik et al.	11
US20170281091A1	Capsule device and methodology for discovery of gut microbe roles in diseases with origin in gut	Lowell Zane Shuck	12
US20170372027A1	Method and system for microbiome-derived diagnostics and therapeutics for locomotor system conditions	uBiome Inc.	14
US20170286620A1	Method and system for microbiome-derived diagnostics and therapeutics	uBiome Inc.	14
US20190030095A1	Methods and compositions relating to microbial treatment and diagnosis of disorders	Whole Biome Inc.	14
WO2017216820A1	Metagenomic method *for in vitro* diagnosis of gut dysbiosis	Putignani et al.	16
WO2017171563A1	Beta-caseins and cognitive function	Clarke et al.	16
WO2017160711A1	Modulation of the gut microbiome to treat mental disorders or diseases of the central nervous system	Strandwitz et al.	17
US20180318323A1	Compositions and methods for improving gut health	Plexus Worldwide LLC	17

**TABLE 6:** Glossary of terms.

**Alpha diversity.** A measure that quantifies the species diversity in a given sample. It can be calculated by several methods including richness (i.e. the number of unique species) as well as the Shannon index which relies on the relative abundance of unique species.	**Marker gene sequencing.** Primer-based strategy (such as 16S rRNA) that targets a specific region of a gene of interest to characterize microbial phylogenies of a sample.
**Beta diversity.** A measure that quantifies the difference between species abundances across samples. It can be calculated by several methods including the Jaccard index (i.e. the ratio of shared to total unique species in a pair of samples) as well as the weighted Jaccard index which also considers the number of times each specie is observed.	**Multiple-hypothesis testing.** A problem that arises in tests of statistical significance when applied multiple times using different hypotheses.
**Classification.** A type of supervised learning problem where the dependent variables are categorical.	**Overfitting.** A problem that arises in machine learning where parameter values of a model are too closely fit for training data and therefore not useful in practice.
**Cluster analysis.** Unsupervised learning methodology to identify groups of similar datapoints automatically.	**Rarefaction.** A bias correction technique used to enable comparison of diversity measures between communities with unequal sample sizes.
**Collaborative filtering.** Recommendation system methodology which relies on similarities amongst user preferences for new recommendations.	**Recommendation system.** “Any system that guides a user in a personalized way to interesting or useful objects in a large space of possible options or that produces such objects as output.” (Burke, 2002) **Regression.** Supervised learning tasks in which the dependent variables are numerical.
**Compositional quantities.** Dataset attributes that their absolute quantities are only meaningful relative to each other for each sample, and cannot be compared directly across different samples.	**Regularization.** Machine learning technique that dampens the variability of model parameters leading to a less complex model. It is usually used to mitigate overfitting.
**Content-based filtering.** Recommendation system methodology in which recommendations are made based on the features for both items and users.	**Stability metric.** A quantitative measure to assess whether properties of a community (e.g., gut microbes) are preserved over time.
**Curse of dimensionality.** A set of challenges, such as the need of exponentially more samples to train a model and increased computational complexity, that appear when the dimensionality of the data or model increases.	**Supervised learning.** Learning tasks that require labeled data. They involve learning a function to predict the correct label for a new sample given input attributes.
**Data imputation.** Substitution of missing values in a given dataset.	**Unsupervised learning.** Learning tasks that do not rely on labeled data. They involve learning hidden structures, features, or patterns within the data.
**Diversity metric.** Quantitative measure that represents the number of unique entity types (e.g., species) in a community and evenness in their relative population.	**Variation analysis.** Statistical methods, such as analysis of variance (ANOVA), used to identify the amount of variance in a dependent variable that can be explained using independent variables.
**Dimensionality.** Number of attributes available for each sample in a given dataset. A dataset with relatively few attributes is considered *low-dimensional* while a dataset with many attributes is referred to as *high-dimensional*.
**Labeled/unlabeled samples.** Samples that have been tagged using particular labels describing the value of a dependent variable are called *labeled*. This is in contrast to *unlabeled* samples for which such labels are unavailable. Note that labels can be categorical or numerical.	**Whole metagenomic sequencing.** A sequencing strategy that targets the whole genome of all microbial species within a sample. This is also called shotgun metagenomics.

Even though there is already a considerable number of patent applications for technologies aiming to manipulate the gut microbiome for multiple health conditions, regulatory legislation has not yet become specific to deal with the new scientific advances in the field. In Europe, the European Food Safety Authority (EFSA) is responsible for regulating and approving food products with health claims, including probiotics, while in the United States, the Food and Drug Administration (FDA) assumes a similar role. Legislation and regulatory aspects are changing in an attempt to keep up with the ever-evolving field. Recently, the FDA has released a statement (Food and Drug Administration, 2018) clarifying existing regulations and announcing the intention to work closely with the United States National Institutes of Health to ensure public safety. Currently, there is no probiotic approved to be marketed in the United States as a live biotherapeutic product, defined by the agency as a “biological product other than a vaccine that contains live organisms used to prevent or treat a disease or condition in humans” (Food and Drug Administration, 2016,2018). This means that, even though probiotics are legally available as dietary supplements or food ingredients, they cannot yet have claims to cure, treat, or prevent any diseases per current regulation (Food and Drug Administration, 2018), since those claims are reserved for drugs. Classification of food ingredients targeting the microbiome, but not composed of living organisms, microbiota-directed foods or MDFs, prebiotics, and dietary fiber, is also challenging based on the available legislation. Depending on the health claims, such products can fall under the categories of drugs or dietary supplements, which have different requirements for approval (Green et al., 2017).

## Conclusion

Significant advances in microbiology, genomics, analytical chemistry, computational science, bioinformatics, and other critical disciplines have begun to converge such that it is possible to foresee a new era of health and nutrition research enabling the design of food products capable of optimizing health via predictable interactions with the gut microbiome. Despite the exciting potential in this context demonstrated by pioneering research efforts of many investigators, including those cited in this brief review, the complexity of the microbiome, the chemical composition of food, and their interplay *in situ* remains a daunting challenge in the context of achieving necessary breakthroughs. However, recent advances in high-throughput sequencing and metabolomics profiling, compositional analysis of food, and the emergence of electronic health records as an opportunity to integrate health information provide new sources of data that can contribute to addressing this challenge. Indeed, it is now clear that computational science will play an essential role in this effort as it will provide the foundation to integrate these data layers and derive insights capable of revealing and understanding the complex interactions between diet, microbiome, and health.

The human microbiome is exceptionally plastic, which presents both challenges and opportunities (Gentile and Weir, 2018). Due to its temporal and inter-individual variability, it is difficult to discover statistically significant signatures that unambiguously constitute a healthy versus non-healthy microbiota. At the same time, its potential for adaptation to diet and other environmental factors makes the gut microbiome an excellent target for diet-related interventions to improve health. In this article, we presented a brief overview of the current state of knowledge and potential avenues for research at the interface of diet, gut microbiome, and human health, with particular emphasis on the role that computational science and data analytics can play in accelerating this research. Using these tools, we envision a future in which diets, as well as food and dietary supplement products, can be better designed for specific populations, and, in some cases, for individuals, in order to optimize gut microbiota and health via a platform integrating two distinct systems. The first system will be responsible for identifying the optimal target microbiota (*discovery*) given the desired target, individual, and environment, while the second will provide recommendations for achieving that target microbiota (*engineering*). Recognizing this distinction and the requirement for seamless interaction between the two can reinforce collaborative research in this evolving field where some teams focus on microbiota discovery and others on diet engineering.

Microbiome research has attracted much interest in the past few years and given rise to various software tools and pipelines for metagenomic data processing and analysis. Many of these tools address similar problems and researchers may choose a variety of tools depending on the context. Interestingly, recent research has shown that synthetic datasets can be used to assess the performance of competing tools given a project’s assumptions and hence provide useful benchmarks (Ounit and Lonardi, 2016; Hitch and Creevey, 2018). We further believe that progress in simulation-based studies can give rise to new data processing and analytics pipelines customized for each project based on factors such as sequencing technology, data availability, dimensionality, and variability. This can help to build standard protocols for addressing challenges like the ones mentioned in Tables 3, 4.

Our current knowledge about the relationship between diet, gut microbiome, and human health is evolving fast. Many data analysis methods exist for discovering characteristics that can define a healthy microbiota and the factors influencing it. We believe that proper integration of recommendation systems with existing research developments will have an unprecedented impact on our way of life. Given the accelerated pace of advances in sequencing and computational tools, we expect the next decade to be the era of computational nutrition that will revolutionize our relationship with food and diet.

## Author Contributions

AE, NR, BP, MK, HS, and IT wrote the manuscript. AE and MK created figures with input from all authors. IT supervised all aspects of the work. All authors reviewed, revised, and approved the manuscript.

## Conflict of Interest

MK and IT are employed or have a financial interest in PIPA LLC. HS has a financial interest in T.O.P. LLC and March Capital US LLC. The remaining authors declare that the research was conducted in the absence of any commercial or financial relationships that could be construed as a potential conflict of interest.
